# Anaplastic Large Cell Lymphoma in Children: 20-Year Immune-Oriented Treatment Experience

**DOI:** 10.3390/cancers18101583

**Published:** 2026-05-13

**Authors:** Anastasiya S. Volkova, Timur T. Valiev, Mikhail V. Kiselevskiy, Irina Zh. Shubina, Kirill I. Kirgizov, Svetlana R. Varfolomeeva, Ivan S. Stilidi

**Affiliations:** 1 Research Institute of Pediatric Oncology and Hematology, FSBI “N.N. Blokhin National Medical Research Center of Oncology” of the Ministry of Health of Russia, Kashirskoye sh. 23, Moscow 115522, Russia; a.s.volkova@ronc.ru (A.S.V.); t.valiev@ronc.ru (T.T.V.); k.kirgizov@ronc.ru (K.I.K.); s.varfolomeeva@ronc.ru (S.R.V.); 2 Research Institute of Experimental Oncology and Carcinogenesis, FSBI “N.N. Blokhin National Medical Research Center of Oncology” of the Ministry of Health of Russia, Kashirskoye sh. 24, Moscow 115522, Russia; kisele@inbox.ru; 3 Management Department, FSBI “N.N. Blokhin National Medical Research Center of Oncology” of the Ministry of Health of Russia, Kashirskoye sh. 24, Moscow 115522, Russia; director@ronc.ru

**Keywords:** anaplastic large cell lymphoma, pediatric lymphoma, immunophenotype, chemotherapy, T-cell markers, NHL-BFM 95, ALCL NII DOiG 2003, survival analysis

## Abstract

**Background**: Anaplastic large cell lymphoma (ALCL) accounts for up to 15% of all pediatric and adolescent non-Hodgkin lymphomas and is characterized by significant clinical, morphological, and immunohistochemical heterogeneity. Expression of T-cell markers on tumor cells is considered as one of the factors in an unfavorable prognosis in ALCL. However, no treatment protocols based on ALCL immunological heterogeneity have been used, yet. **Objective**: To compare the effectiveness of immunophenotype-oriented chemotherapy in children with ALCL treated according to the ALCL NII DOiG 2003 protocol versus the standard NHL-BFM 95 protocol. **Methods**: A retrospective–prospective analysis of 100 newly diagnosed ALCL patients, who were treated between 2000 and 2023, was performed across five pediatric oncology and hematology centers in Russia. Patients were divided into two groups: those treated with the NHL-BFM 95 protocol (*n* = 52) and those treated with the ALCL NII DOiG 2003 protocol (*n* = 48). Comparative analysis used Kaplan–Meier survival curves constructed for each group, and statistical analysis was performed with IBM SPSS Statistics 21.0. **Results**: The 10-year overall survival was significantly higher in the ALCL NII DOiG 2003 group (95.3 ± 3.3%) compared to that of the NHL-BFM 95 group (82.0 ± 5.4%, *p* = 0.037). Event-free survival was also improved (95.3 ± 3.3% vs. 68.6 ± 6.5%, *p* = 0.001), as well as the relapse-free survival (97.3 ± 2.7% vs. 74.4 ± 6.4%, *p* = 0.003). **Conclusions**: The immunophenotype-oriented approach of the ALCL NII DOiG 2003 protocol provides significantly improved long-term outcomes compared to the common NHL-BFM 95. These findings support the benefit of personalized immunologically targeted therapy in pediatric ALCL treatment.

## Simple Summary

Anaplastic large cell lymphoma (ALCL) accounts for up to 15% of all pediatric and adolescent non-Hodgkin lymphomas and is characterized by significant clinical, morphological, and immunohistochemical heterogeneity. Along with an advanced stage and involvement of the skin, bones, and lungs, one of the factors for an unfavorable prognosis is the expression of T-cell markers by tumor cells. We designed an ALCL NII DOiG 2003 treatment protocol based on immunological heterogeneity and compared its efficacy with that of the standard NHL-BFM 95 protocol. A retrospective–prospective analysis included 100 newly diagnosed ALCL patients who were treated between 2000 and 2023 across five pediatric oncology and hematology centers in Russia. Our study confirmed that the immunophenotype-oriented approach led to significantly improved 10-year overall survival (95.3% vs. 82.0%), event-free survival (95.3% vs. 68.6%), and relapse-free survival (97.3% vs. 74.4%). These findings support the benefit of immunologically stratified chemotherapy for pediatric anaplastic large cell lymphoma.

## 1. Introduction

Anaplastic large cell lymphoma (ALCL) accounts for up to 15% of non-Hodgkin lymphoma (NHL) cases among children and adolescents, making it the third most common subtype. In the adult NHL population, ALCL is diagnosed less frequently—up to 3% of cases only. The median age at diagnosis is 11–12 years, and boys present with it 3–5 times more often than girls [1,2,3].

According to the 2016 WHO classification of tumors originating from hematopoietic and lymphoid tissues, ALCL is divided into four main types: ALK-positive (ALK+), ALK-negative (ALK−) (ALK—anaplastic lymphoma kinase), primary cutaneous ALCL, and breast implant-associated ALCL. In pediatric practice, the ALK+ variant represents more than 95% of ALCL cases. ALK−ALCL is observed primarily in adult patients over 40 years and includes subtypes with *DUSP22* or *TP63* rearrangements and cases with no detectable genetic abnormalities [4,5,6].

ALCL is characterized by morphological heterogeneity; five main histological subtypes are recognized: common, small cell, lymphohistiocytic, Hodgkin-like, and composite (mixed). The common subtype is the most frequent (60–70%), while the small cell and lymphohistiocytic subtypes each account for up to 10%. The Hodgkin-like subtype represents about 3% of cases. Rare histological variants (1%) include the hypocellular and sarcomatoid variants. In 15% of cases, histological analysis reveals features typical of multiple subtypes—referred to as the composite (mixed) variant of ALCL [4,5,6,7,8].

The immunohistochemical profile of ALCL is defined primarily by CD30 expression, which is detected in 100% of cases (Table 1). It should be noted, however, that CD30 is not strictly specific to ALCL, as its expression is also observed in other diseases, including Hodgkin lymphoma.

Anaplastic large cell lymphoma is characterized by a specific chromosomal translocation, t(2;5)(p23;q35), resulting from the fusion of ALK (on chromosome 2) with NPM (on chromosome 5). Formation of the chimeric NPM–ALK transcript leads to the activation of multiple signaling pathways regulating cell proliferation and apoptosis, including RAS/RAF/MEK/ERK1/2, JAK/STAT, and PI3K/AKT. In 15–20% of cases, alternative chromosomal translocations may occur, such as:

- t(1;2)(q21;p23) (TPM3–ALK fusion),
- inv(2)(p23;q35) (ATIC–ALK),
- t(2;3)(p23;q21) (TFG–ALK),
- t(2;19)(p23;q13.1) (TPM4–ALK),
- t(2;X)(p23;q11–12) (MSN–ALK),
- t(2;17)(p23;q23) (CLTCL–ALK) [8,9,10,11].

In 90–95% of ALCL cases, ALK protein expression is detected, correlating with chromosomal rearrangements involving this gene. Since ALK+ and ALK− ALCL are morphologically indistinguishable, immunohistochemical assessment of ALK expression is crucial. Although ALK is a highly sensitive marker for ALCL, it is not specific for this disease; ALK expression was reported in lung carcinoma, other solid tumors, and ALK+ diffuse large B-cell lymphoma [6,7,8]. ALCL demonstrates variable expression of T-cell markers. According to Muzzafar et al., CD2 is expressed in 67% of cases, CD7 in 60%, CD3 in 45%, CD4 in 33%, CD5 in 14%, and CD8 in 14%. Approximately 20% of patients have a “null” immunophenotype (no T-cell antigens on tumor cells); however, clonal rearrangement of T-cell receptor genes is almost always detected, confirming the T-lineage origin of ALCL [10,11,3,12,13,14].

Several studies showed a negative prognostic impact of T-cell marker expression. In 2013, Abramov et al. reported significantly lower survival rates in CD8+ ALCL patients: 5-year event-free survival (EFS) was 68 ± 5% in CD8− ALCL patients and 25 ± 10% in CD8+ patients (*p* < 0.001); 5-year overall survival (OS) was 84% and 55%, respectively [15]. Furthermore, Mussolin et al. demonstrated an unfavorable prognosis associated with CD3 expression: 10-year progression-free survival in CD3+ ALCL patients was 56%, compared to 74% in CD3− patients [16]. However, Hapgood et al. showed that CD3 expression was more common in ALK− ALCL than in ALK+ ALCL, which makes the connection between CD3+ and poor outcomes ambiguous [13]. A recent prospective clinical study including 89 newly diagnosed pediatric ALCL patients demonstrated that CD3+ was associated with the central nervous system (CNS) disorders; however, there were no significant differences between CD3+ and CD3− patients in EFS, relapse-free survival (RFS), or OS [17].

Clinical manifestations of ALCL in children are diverse. At the time of diagnosis, the disease is usually registered as an advanced process (stages III–IV), which involves lymph nodes and different extranodal sites (skin, soft tissue, liver, bone, bone marrow, and rarely, the CNS). Most patients have persistent lymphadenopathy and B-cell symptoms at onset. In rare cases, lymphadenopathy may be intermittent, which can delay diagnosis. Additionally, the reports describe cases of ALCL with clinical features resembling acute leukemia due to bone marrow involvement, particularly in the small-cell lymphoma variant [18,19].

Different treatment approaches for ALCL have been designed and introduced in clinical settings over the past 30 years. For instance, the Berlin–Frankfurt–Münster (BFM) group employed short-term intensive regimens similar to those used for mature B-cell NHL. The regimens were typically based on the predominant use of alkylating agents with minimum anthracyclines [20]. In contrast, the Children’s Cancer Group (CCG), Pediatric Oncology Group (POG), and Children’s Oncology Group (COG) used prolonged (acute lymphoblastic leukemia (ALL)-focused) treatment programs. These protocols included anthracyclines in higher cumulative doses, while the doses of alkylating agents were minimal [21,22,23]. Regardless of the treatment approach, the studies achieved comparable survival rates. Thus, none of the treatment strategies demonstrated a marked advantage over the other regimens. Despite rather satisfactory OS and EFS rates, relapses occurred in 25–30% of patients within the first year after completion of any therapy used.

The development of targeted drugs has opened new opportunities for ALCL treatment. In most cases (over 90%), children have ALK+ ALCL, which implies the use of drugs that inhibit ALK phosphorylation and prevent tumor cell proliferation. Crizotinib, a first-generation ALK inhibitor, was approved by the FDA in 2021 for the treatment of relapsed ALK+ ALCL. A clinical trial conducted by the COG, which included 26 patients aged 1 to 22 years with relapsed ALK+ ALCL, demonstrated that Crizotinib monotherapy achieved an objective response rate (ORR) of 90% [24]. At the same time, Crizotinib in the first-line therapy did not lead to a significant improvement in survival. One of the most recent COG trials, ANHL12P1, performed between 2013 and 2017, aimed to evaluate the efficacy and tolerability of Brentuximab vedotin and Crizotinib as part of the first-line therapy for ALCL. In the second arm of the ANHL12P1 protocol, patients (n = 66) with advanced ALCL received the first-generation ALK inhibitor Crizotinib as part of combination chemotherapy. The 2-year EFS was 76.8%, and the 2-year OS was 95.2%. Fifteen patients had relapses, with a median time to relapse of 7.4 months from diagnosis [25,26]. Next-generation ALK inhibitors, including Alectinib, Ceritinib, Brigatinib, Lorlatinib, and Crizotinib, were more effective than Crizotinib and showed better CNS penetration. However, treatment with ALK inhibitors required long-term administration and had a high risk of relapse after therapy discontinuation [27].

ALCL cells also express CD30, which can be targeted by a drug. Brentuximab vedotin is an antibody–conjugate drug, which consists of a monoclonal antibody against CD30 linked to the antimitotic agent monomethyl auristatin E. Earlier some authors reported Brentuximab vedotin-based therapy for pediatric patients with refractory or relapsed ALCL. Locatelli et al. described Brentuximab vedotin monotherapy in 17 patients with refractory or relapsed ALCL. The overall response rate was 53%, with complete remission achieved in 41% and partial remission in 12% of cases [28]. First time Brentuximab vedotin was used for the first-line treatment of ALCL in the ANHL12P1 study. The treatment protocol included a pre-phase followed by six cycles of polychemotherapy (based on the ALCL99 regimen) with Brentuximab vedotin administration on day 1 of the cycle. The planned dose was 1.8 mg/kg (maximum 180 mg) and included possible dose reductions in cases of toxicity. A total of 14 out of 68 patients developed a relapse. Relapse was registered within 10 months from the start of the treatment in 79% of those cases. The 2-year EFS was 79.1%, and the 2-year OS was 97.0% [25].

PD-L1 is also expressed by ALCL cells, indicating the potential of immune checkpoint inhibitors (ICIs) in the treatment of patients with ALCL.

However, a phase II clinical trial of nivolumab in refractory or relapsed forms of T-cell lymphomas showed disappointing results. The efficacy of ICI therapy was only 33%, and cases of hyperprogression were observed, apparently due to the activation of transformed T-lymphoma cells [29]. At the same time, several small clinical studies and individual case reports described the effective use of nivolumab or pembrolizumab in patients with refractory or relapsed ALCL, suggesting that ICI therapy may be effective in treating relapsed ALCL even in the absence of PD-L1 expression on tumor cells [30,31]. The use of ICIs in patients after allogeneic hematopoietic stem cell transplantation (allo-HSCT) is limited due to severe adverse events, which in some cases led to treatment discontinuation [32]. At the same time, it should be noted that the use of ICIs after allo-HSCT enhances the severity of graft-versus-host disease (GVHD).

On the other hand, according to Baermann et al., GVHD is associated with long-term remission after treatment and the graft-versus-lymphoma effect induced by nivolumab may be more important than the PD-1 blockade [33].

Further efforts should be made to conduct prospective studies aimed at determining the maximum tolerated dose and optimal duration of therapy.

CAR-T cell therapy has demonstrated high efficacy in B-cell lymphomas, but the results of clinical trials of CAR-T cells in T-cell lymphomas, including ALCL, were highly contradictory. The transmembrane receptor CD30 was overexpressed in ALCL cells, while its expression in normal cells was limited. Clinical studies evaluating the efficacy and safety of anti-CD30 CAR-T cell therapy in patients with relapsed/refractory (r/r) CD30+ ALCL showed inconsistent results. For example, in patients with classical ALCL, anti-CD30 CAR-T cells demonstrated transient efficacy and significant toxicity [34]. Several phase I studies of anti-CD30 CAR-T cells in CD30+ Hodgkin lymphoma yielded modest results, with the best response being three complete remissions among nine patients [35,36]. Clinical studies showed that in relapsed or refractory ALCL, high-dose chemotherapy followed by allogeneic (allo-HSCT) or autologous (auto-HSCT) hematopoietic stem cell transplantation could be a treatment option for patients who failed to achieve complete remission with conventional chemotherapy [37].

The German research group defined the indications for stem cell transplantation as follows: allogeneic HSCT was performed in patients with disease progression during treatment or with early CD3-positive relapse of ALCL; autologous HSCT was used in patients with CD3-negative early relapse, as well as in those with late relapse and a history of vinblastine therapy.

Notably, HSCT is considered a therapeutic option only for patients receiving second-line or subsequent therapy.

The International, Prospective ALCL-Relapse Trial, which included 105 patients, demonstrated the efficacy of allo-HSCT in patients with high-risk ALCL relapse. In contrast, auto-HSCT in intermediate-risk patients with early relapse proved ineffective: the 5-year EFS and OS rates were 41% and 82%, respectively. As potential unfavorable prognostic factors in patients with relapse, the authors noted immunological features of the tumor (such as CD3 expression on tumor cell surfaces) and early relapse [38]. Thus, the identification of tactics and possible treatment approaches for patients with r/r ALCL remains an important and urgent task for researchers.

The ALCL NII DOiG 2003 protocol was designed to consider both standard prognostic risk group factors and tumor immunophenotype taking into account a specific role of T-cell receptor as a defining marker of ALCL.

The aim of the present study was to evaluate the efficacy of treatment in children with ALCL according to the differentiated chemotherapy protocol based on ALCL immunophenotype (ALCL NII DOiG 2003) in comparison with the standard NHL-BFM 95 protocol.

## 2. Materials and Methods

From 2000 to 2023, a total of 100 patients with newly diagnosed ALCL were included in the multicenter retrospective–prospective study. Treatment was performed in the Department of Oncology and Hematology of the Research Institute of Pediatric Oncology and Hematology, FSBI “N.N. Blokhin National Medical Research Center of Oncology”, Ministry of Health of Russia, as well as in pediatric oncology centers in Kazan, Yoshkar-Ola, Donetsk, and Baku (Azerbaijan). The study included patients with a newly diagnosed ALCL, aged ≤ 18 years, who had not previously received antitumor treatment, and whose legal representatives signed informed consent to participate in the study.

The key differences in the ALCL NII DOiG protocol included modifications to the chemotherapy for ALCL based on the expression of T-cell markers. If these markers were detected, patients received blocks of polychemotherapy that included L-asparaginase. Furthermore, vincristine was replaced with vinblastine in the block therapy. A key feature of the protocol was maintenance therapy with vinblastine for six months after completion of intensive antitumor treatment. Regardless of T-cell marker expression, patients received vinblastine as part of each block and as part of maintenance therapy.

**Figure 1:**
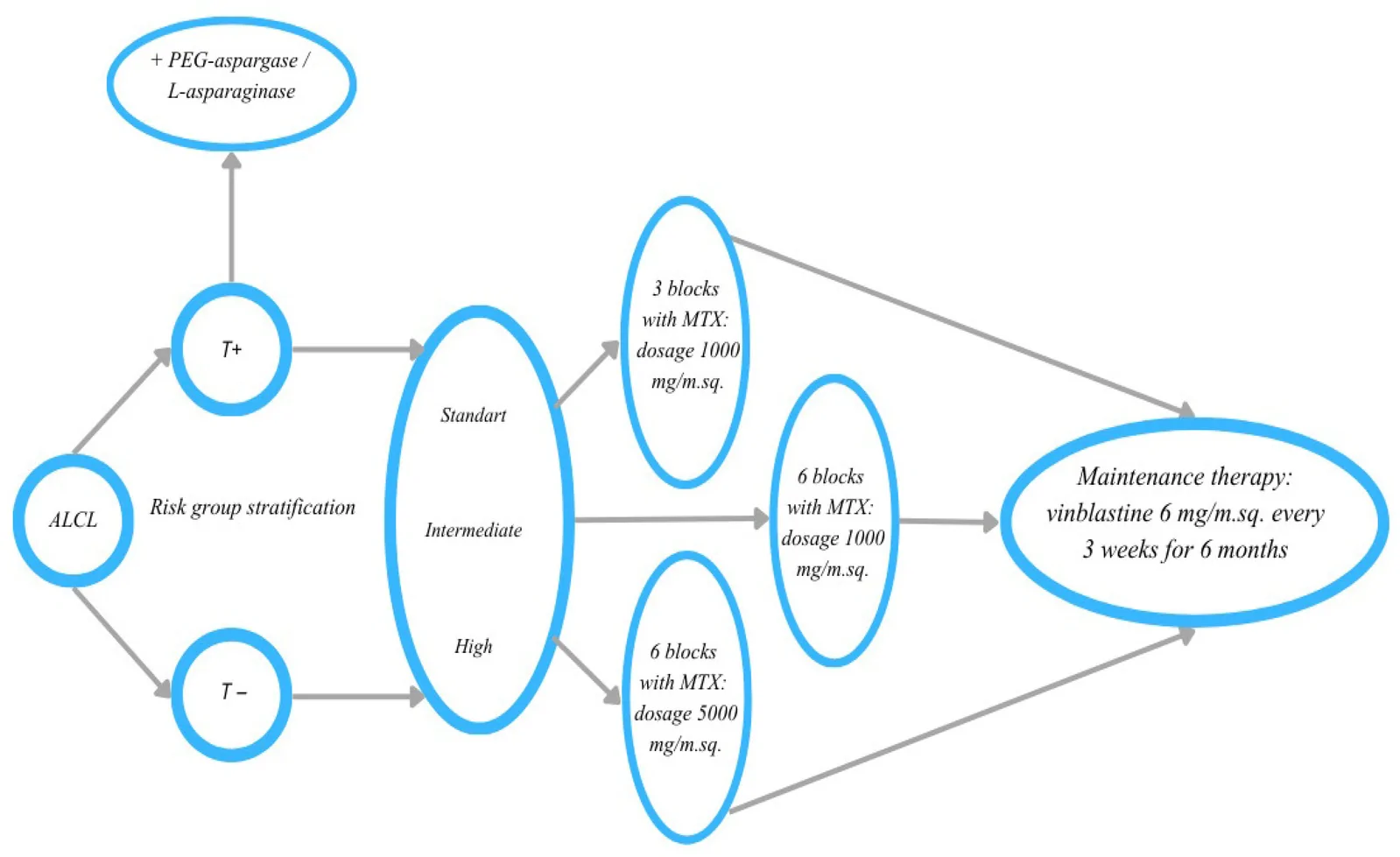
Scheme of the protocol ALCL NII DOiG 2003. Note: If T-cell markers were detected, patients received treatment with the inclusion of block modes similar to those used in high-risk ALL patients. If expression of T-cell markers was not confirmed, block therapy was similar to that used in the NHL-BFM 95 protocol.

Of these patients, 52 received treatment according to the NHL-BFM 95 protocol, and 48 were treated according to the ALCL NII DOiG 2003 protocol (Figure 1). The results of OS, EFS, and RFS were calculated as of 1 September 2023. The study was conducted in accordance with the Declaration of Helsinki and approved by the Institutional Ethics Committee of the FSBI “N.N. Blokhin National Medical Research Center of Oncology” of the Ministry of Health of Russia, Approval protocol of 28 October 2002. Informed consent was obtained from the legal representatives of the patients involved in the study.

The diagnosis of ALCL was established based on histological, immunohistochemical, and cytogenetic studies, in accordance with the 5th Edition of the WHO classification of tumors of hematopoietic and lymphoid tissues.

Immunohistochemical analysis included assessment of ALK, CD30, T-cell markers (CD2, CD3, CD4, CD5, CD7, CD8), Ki-67, TIA1, granzyme B, and perforin expression. Patient staging was performed using the S. Murphy system, while risk group stratification followed the criteria of the BFM group. The following risk groups were identified:

- Standard risk: patients with completely resected tumors and disease Stage I–II.
- Intermediate risk: patients with unresected tumors and disease Stage I–III.
- High risk: patients with multiple lesions of the skin, bones, lungs and/or disease Stage IV.

Treatment Program.

At the initial phase, patients in the intermediate- and high-risk groups received a cytoreductive treatment consisting of:

- Cyclophosphamide 200 mg/m^2^ IV infusion over 1 h on days 1 and 2;
- Dexamethasone 5 mg/m^2^ on days 1–2, then 10 mg/m^2^ on days 3–5;
- Intrathecal administration of methotrexate, cytarabine, and prednisolone on day 1 in age-appropriate doses (Table 2).

Patients in the standard-risk group did not receive cytoreductive therapy.

In cases where T-cell markers were expressed on tumor cells, treatment included high-intensity blocks similar to those used during the consolidation phase for high-risk acute lymphoblastic leukemia (ALL).

In the absence of T-cell marker expression, standard block chemotherapy was administered, corresponding to the NHL-BFM 95 regimen.

After completing the block therapy, patients received vinblastine at a dose of 6 mg/m^2^ (maximum single dose 10 mg) once every three weeks for six months.

Treatment protocol for patients with ALCL without T-cell marker expressions:

- Risk Group I (standard risk): Treatment consisted of 3 blocks (alternating 1a #2, 1b#1) administered every two weeks.
- Risk Group II (intermediate risk): Patients received 6 blocks of polychemotherapy (1a #3, 1b #3) administered at two-week intervals.

**Block 1a:**

- Dexamethasone 10 mg/m^2^ orally, days 1–5;
- Vinblastine 6 mg/m^2^ IV bolus, day 1 (max 10 mg);
- Ifosfamide 800 mg/m^2^ IV over 1 h, with MESNA 300 mg/m^2^ IV at 0, +4, +8 h, days 1–5;
- Etoposide 100 mg/m^2^ IV over 2 h, days 4–5 (2 doses, 24 h apart);
- Cytarabine 150 mg/m^2^ IV over 1 h, days 4–5 (4 doses, 12 h apart);
- Methotrexate 1000 mg/m^2^ IV over 24 h, day 1;
- Leucovorin 15 mg/m^2^ IV at 42, 48, and 54 h from methotrexate start;
- Intrathecal methotrexate, cytarabine, and prednisolone (age-adjusted doses), day 1.

**Block 1b:**

- Dexamethasone 10 mg/m^2^ orally, days 1–5;
- Vinblastine 6 mg/m^2^ IV bolus, day 1 (max 10 mg);
- Cyclophosphamide 200 mg/m^2^ IV over 1 h with MESNA 70 mg/m^2^ IV at 0, +4, +8 h, days 1–5;
- Doxorubicin 25 mg/m^2^ IV over 1 h, days 4–5 (2 doses, 24 h apart);
- Methotrexate 1000 mg/m^2^ IV over 24 h, day 1;
- Leucovorin 15 mg/m^2^ IV at 42, 48, and 54 h after methotrexate start;
- Intrathecal methotrexate, cytarabine, prednisolone, day 1.

Treatment for Risk Group III (high risk):

Same blocks as above with:

- Increased methotrexate dose up to 5000 mg/m^2^;
- 2 additional blocks with high-dose cytarabine.

**Block 3c:**

- Dexamethasone 20 mg/m^2^ orally, days 1–5;
- Vinblastine 6 mg/m^2^ IV bolus, day 1 (max 10 mg);
- Cytarabine 3000 mg/m^2^ IV over 3 h, days 1–2 (4 doses, 12 h apart);
- Etoposide 100 mg/m^2^ IV over 2 h, days 3–5 (5 doses, 12 h apart);
- Intrathecal methotrexate, cytarabine, prednisolone, day 1.

Treatment for patients with ALCL and T-cell marker expressions.

This scheme included:

- High-dose cytarabine (≥2000 mg/m^2^);
- Addition of L-asparaginase at 10,000 IU/m^2^ per block;
- In case of intolerance: pegylated form (Oncaspar) at 1000 IU/m^2^, or 82.5 IU/kg if body surface area < 0.6 m^2^.

Regimen for Risk Group I (T-cell marker positive):

- 3 blocks (Ia, Ib, Ia), every 2 weeks.

**Block Ia:**

- Dexamethasone 10 mg/m^2^ orally, days 1–5;
- Vinblastine 6 mg/m^2^ IV bolus, days 1 and 6 (max 10 mg);
- Methotrexate 1000 mg/m^2^ IV over 24 h, day 1;
- Leucovorin 15 mg/m^2^ IV at 42, 48, 54 h;
- Cyclophosphamide 200 mg/m^2^ IV over 1 h with MESNA 70 mg/m^2^, days 2–4 (5 doses, 12 h apart; first dose 12 h post-MTX);
- Cytarabine 2000 mg/m^2^ IV over 3 h, day 5 (2 doses, 12 h apart);
- L-asparaginase 10,000 IU/m^2^ IV over 2 h, day 6;
- Intrathecal methotrexate, cytarabine, prednisolone, day 1.

**Block Ib:**

- Dexamethasone 10 mg/m^2^ orally, days 1–5;
- Vinblastine 6 mg/m^2^ IV bolus, days 1 and 6 (max 10 mg);
- Ifosfamide 800 mg/m^2^ IV over 1 h with MESNA 300 mg/m^2^, days 2–4 (5 doses, 12 h apart; first dose 12 h post-MTX);
- Daunorubicin 30 mg/m^2^ IV over 24 h, day 5;
- Methotrexate 1000 mg/m^2^ IV over 24 h, day 1;
- Leucovorin 15 mg/m^2^ IV at 42, 48, and 54 h;
- L-asparaginase 10,000 IU/m^2^ IV over 2 h, day 6;
- Intrathecal methotrexate, cytarabine, prednisolone, day 1.

Regimen for Risk Group III (T-cell marker positive):

- Same blocks as above;
- Methotrexate dose increased up to 5000 mg/m^2^ (3a2, 3b2);
- Plus 2 blocks with high-dose cytarabine.

**Block IIIc:**

- Dexamethasone 20 mg/m^2^ orally, days 1–5;
- Vinblastine 6 mg/m^2^ IV bolus, day 1 (max 10 mg);
- Cytarabine 3000 mg/m^2^ IV over 3 h, days 1–2 (4 doses, 12 h apart);
- Etoposide 100 mg/m^2^ IV over 2 h, days 3–5 (5 doses, 12 h apart);
- L-asparaginase 10,000 IU/m^2^ IV over 2 h, day 6;
- Intrathecal methotrexate, cytarabine, prednisolone, day 1.

In the treatment, the following products were used: Dexamethasone (JSC Bryntsalov-A, Elektrogorsk, Russia), Vinblastine (VEROPHARM LLC, Volginsky, Russia), Ifosfamide (JSC Pharmsintez-Nord, St. Petersburg, Russia), MESNA (FSUE “ENDOPHARM”, Moscow, Russia), Etoposide (Rus-Med Exports Private Limited, Gajularamaram, India), Cytarabine (Rus-Med Exports Private Limited, Gajularamaram, India), Methotrexate (VEROPHARM LLC, Volginsky, Russia), Leucovorin (SOOO “Lekpharm”, Logoisk, Republic of Belarus), Intrathecal methotrexate (JSC Pharmsintez-Nord, St. Petersburg, Russia), Prednisolone (LLC “Ellara”, Pokrov, Russia), Cyclophosphamide (JSC Pharmsintez-Nord, St. Petersburg, Russia), L-asparaginase (VEROPHARM LLC, Volginsky, Russia), Oncaspar (Servier Industries Laboratories, Gidy, France).

The analysis of the patients’ data was based on both retrospective and prospective evaluation. The compiled database included individual characteristics of pediatric patients treated under the ALCL NII DOiG 2003 protocol (from 2003 to 2023) and the NHL-BFM 95 protocol (from 1994 to 2023). A comprehensive evaluation of the clinical, morphological, and immunological characteristics of the lymphomas was performed. Treatment efficacy for ALCL was assessed using patient survival statistics and the collected data from both groups.

### Statistical Analysis

Statistical analysis was performed using IBM SPSS Statistics (version 21). Survival outcomes were assessed using the log-rank test. Parametric data were analyzed with Student’s *t*-test, and non-parametric data were compared using Pearson’s chi-square (χ^2^) test based on contingency tables. Kaplan–Meier survival curves were constructed, and differences were considered statistically significant at a *p*-value < 0.05.

## 3. Results

The clinical characteristics of the patients of both treatment protocols were comparable in terms of age, sex, and disease stage (Table 3).

**Figure 2:**
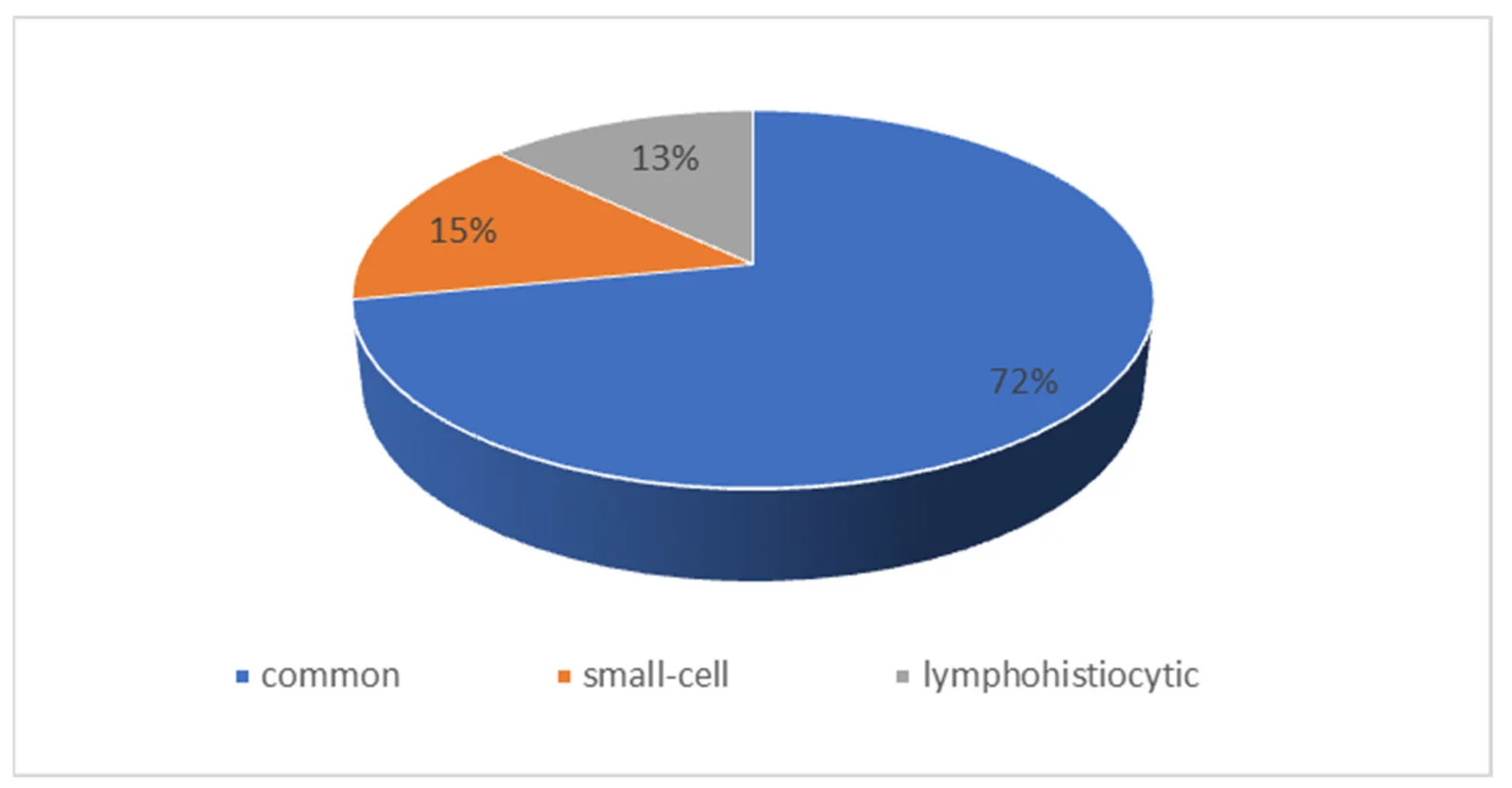
Histological subtypes of ALCL in the study.

**Figure 3:**
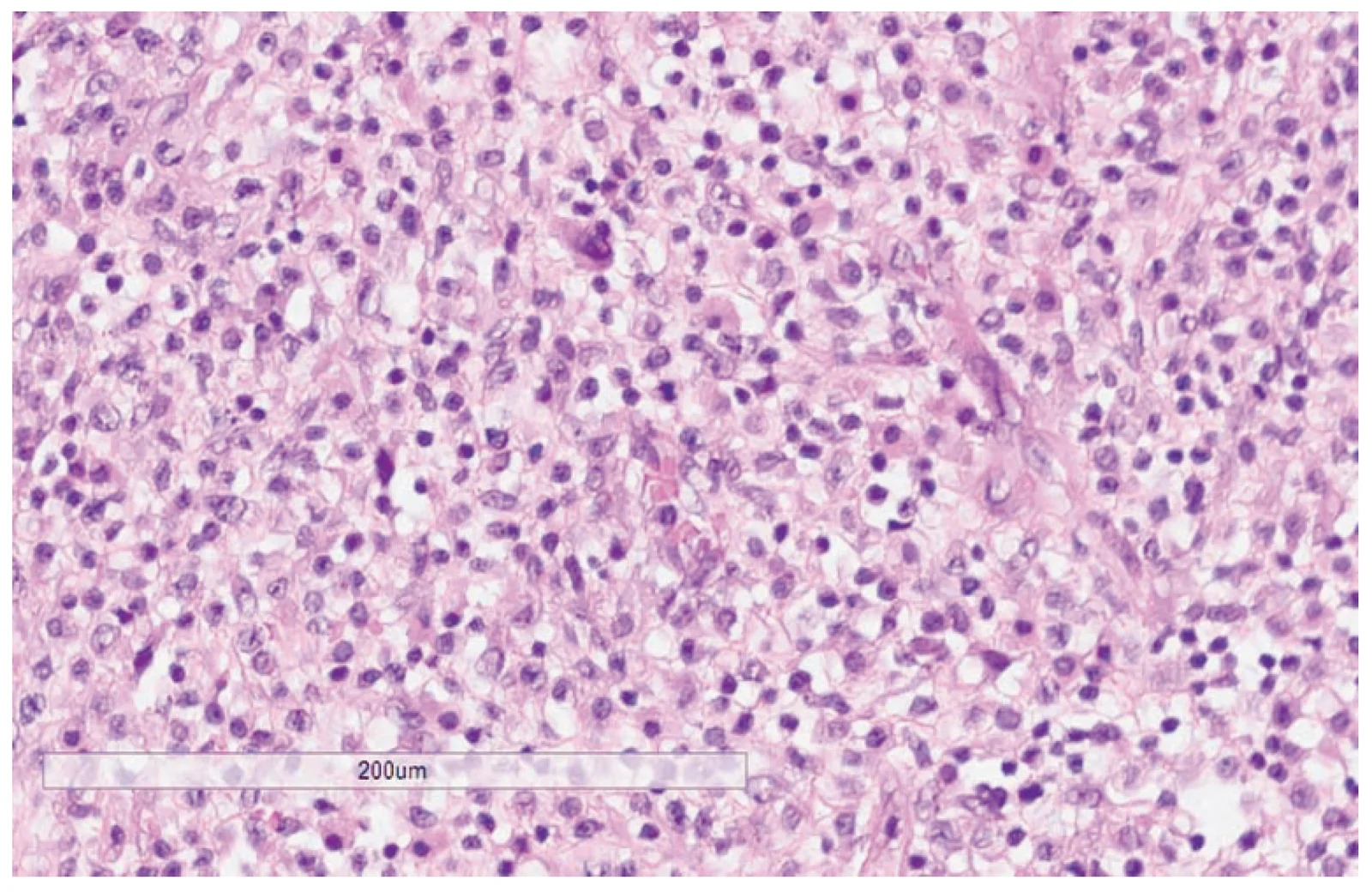
Anaplastic large cell lymphoma: small cell variant. Staining with hematoxylin and eosin. ×200.

**Figure 4:**
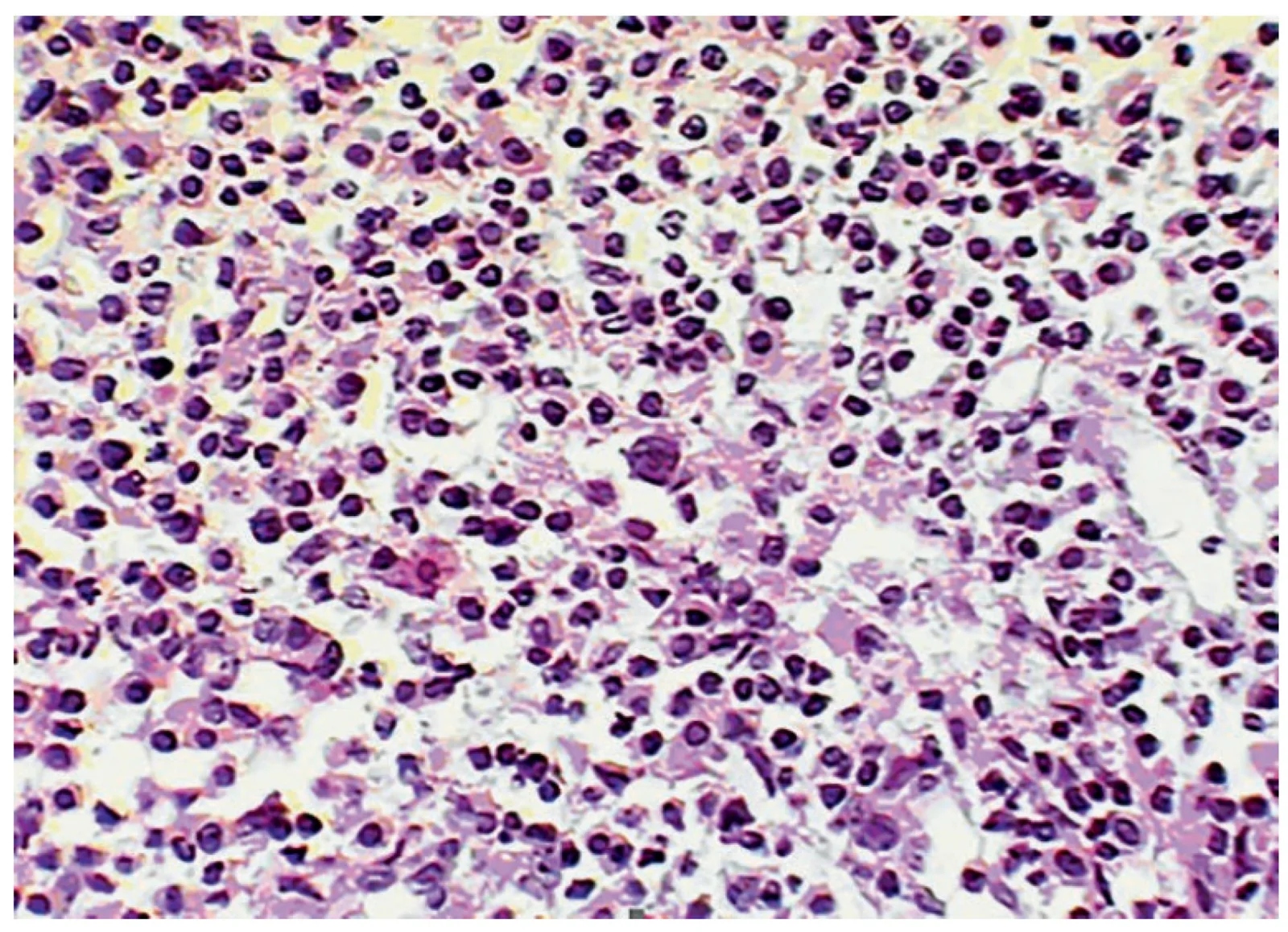
Lymphohistiocytic variant of anaplastic large cell lymphoma. Staining with hematoxylin and eosin. ×200.

**Figure 5:**
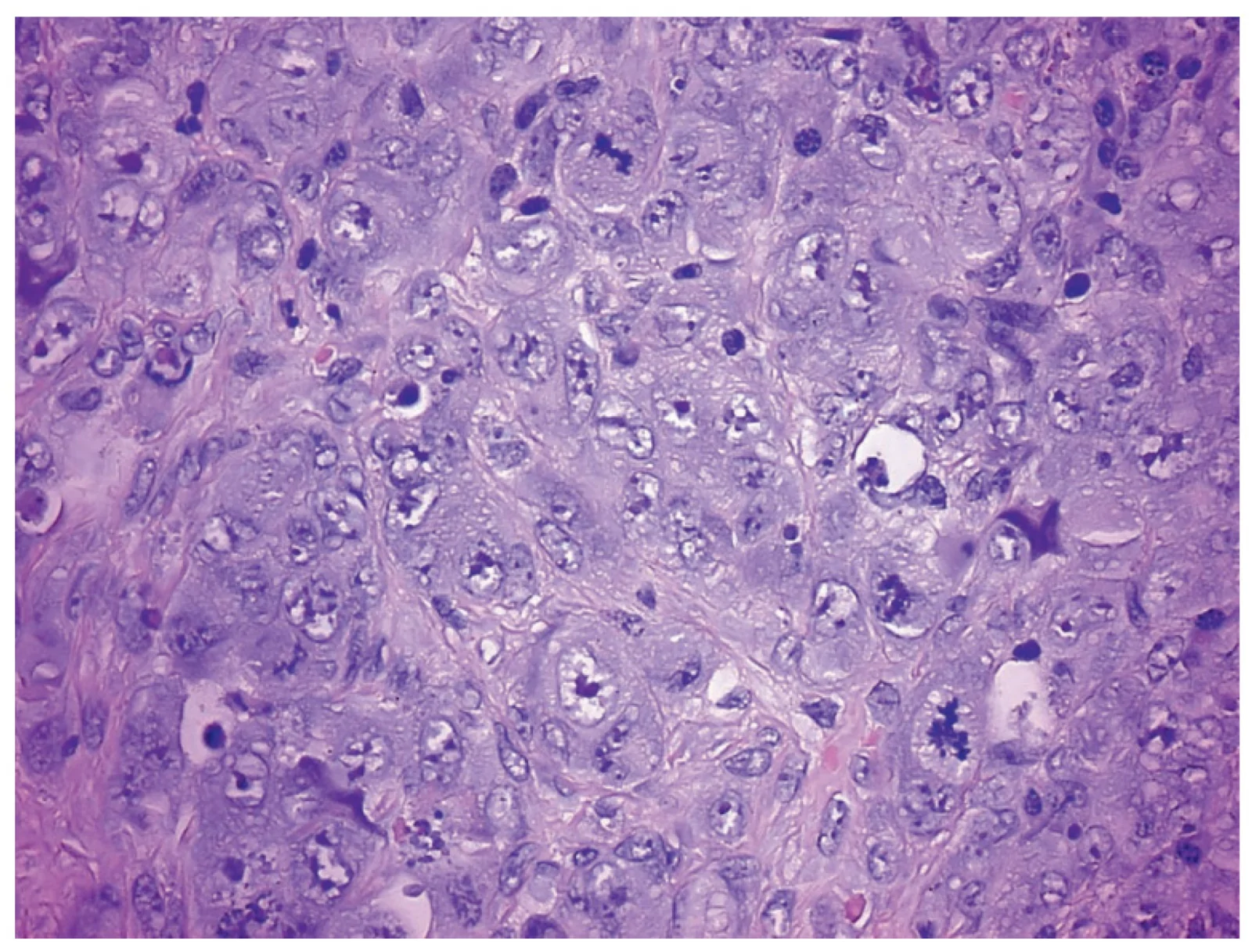
Anaplastic large cell lymphoma: morphological common variant. Staining with hematoxylin and eosin. ×400.

Morphological and immunohistochemical analysis revealed that the most common histological subtype of ALCL among patients included in the current study was the common type (72%), while small-cell and lymphohistiocytic types were observed four times less frequently (Figure 2, Figure 3, Figure 4 and Figure 5). Sarcomatoid, Hodgkin-like, and pleomorphic variants described in the literature were not identified in this cohort.

According to the immunohistochemical data, expression of T-cell markers was detected in 43 patients (43%); these cases were classified as the T-positive (T^+^) ALCL subtype, of which 16 patients received treatment according to the NHL-BFM 95 protocol, and 27 according to the AKKL Research Institute of DOiG 2003 protocol. In 57 patients (57%), no T-cell marker expression was observed; these were classified as the T-negative (T^−^) subtype.

**Figure 6:**
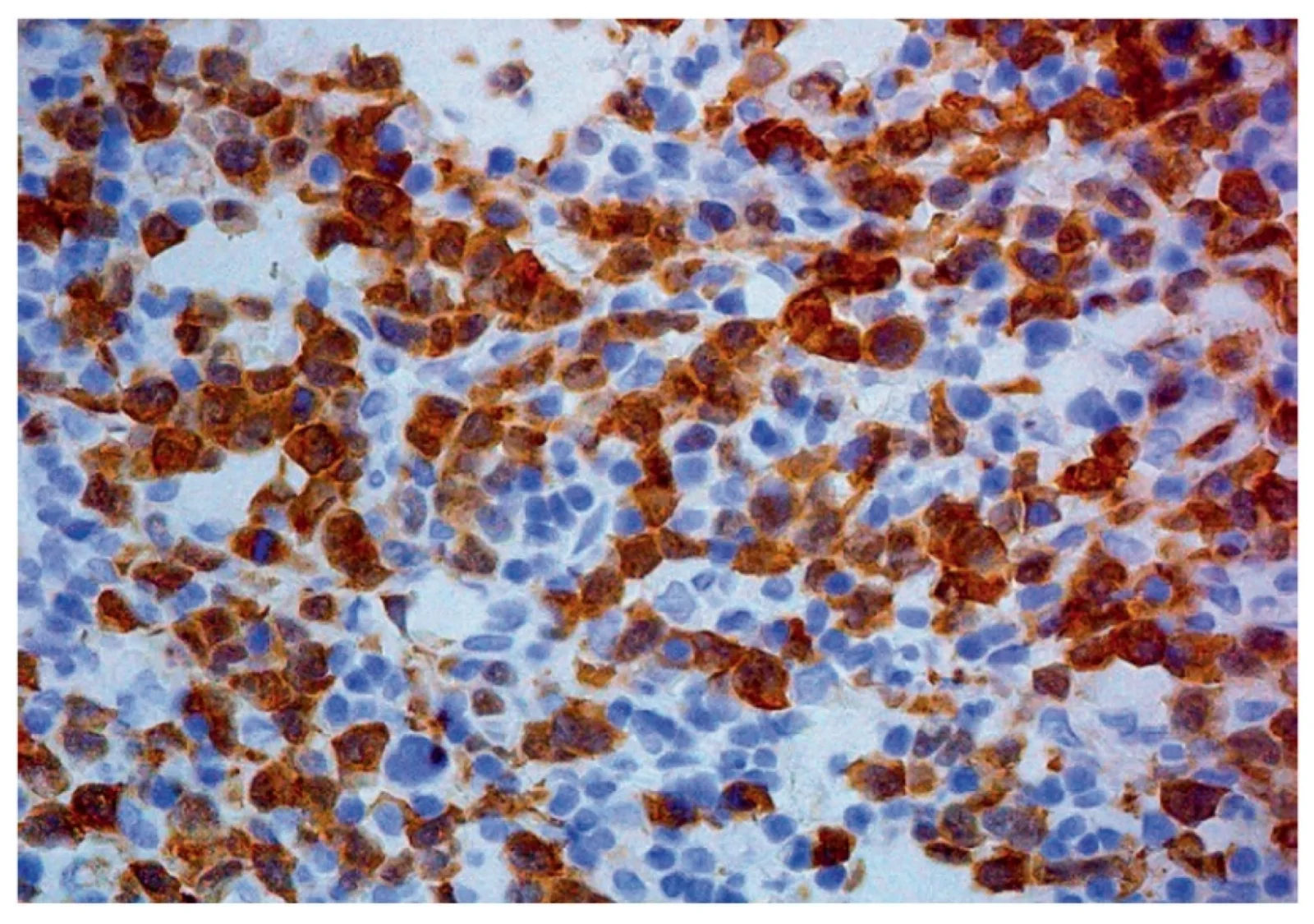
ALK expression in ALCL. The enzyme immunoassay method. ×400.

All patients were tested for ALK protein expression—a recombinant tyrosine kinase protein involved in the malignant transformation of lymphoid cells. Detection of this marker is characteristic of pediatric ALCL and is found in 80–90% of such cases. In this study, ALK expression was identified in 98 patients (98%), while the ALK-negative tumor variant was found in only two patients (2%) (Figure 6).

**Figure 7:**
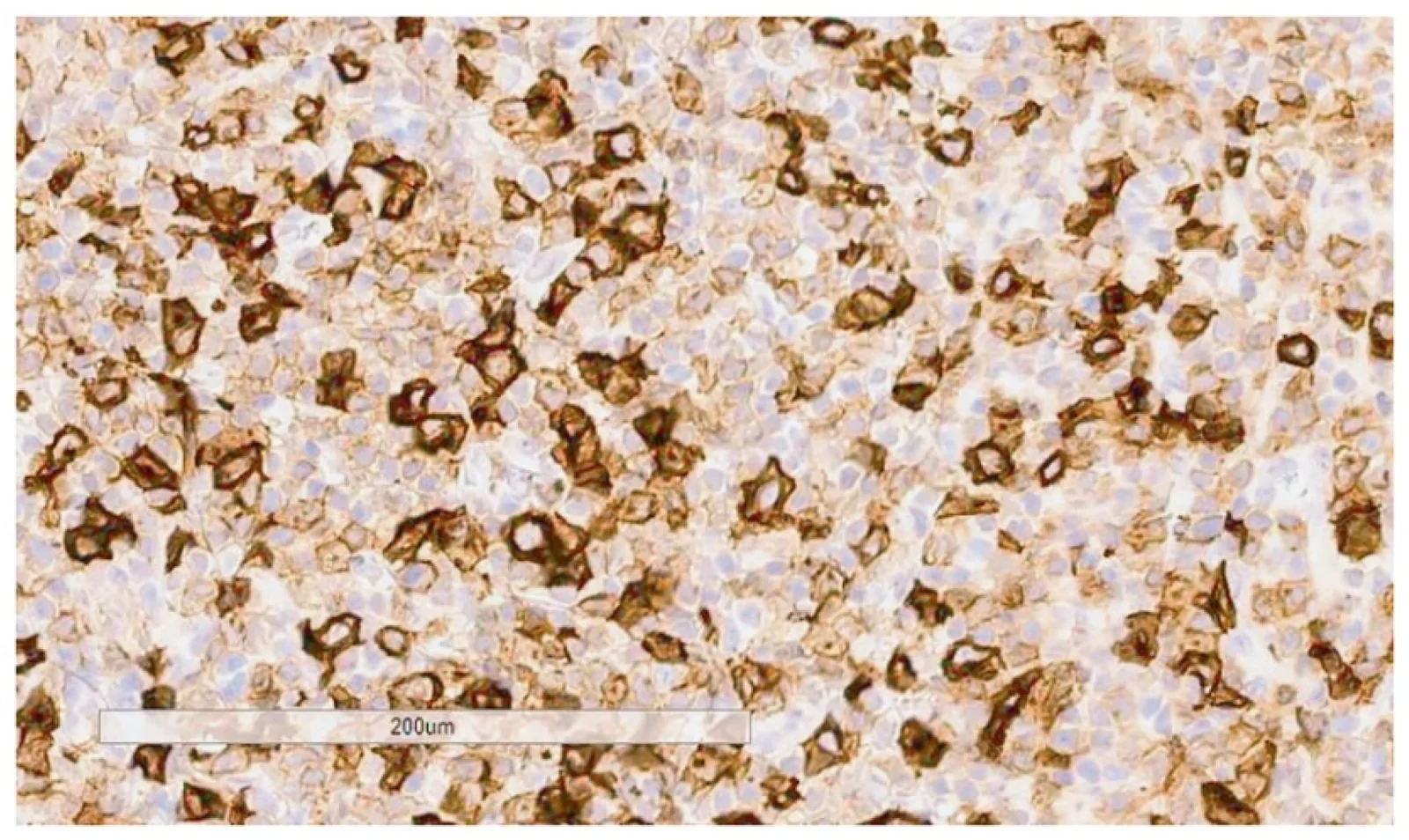
CD30 expression in ALCL. The enzyme immunoassay method. ×400.

CD30 antigen expression was evaluated in all patients. CD30 belongs to the tumor necrosis factor receptor (TNFR) superfamily. Expression of this antigen in ALCL was first described 40 years ago and became one of the earliest defining features of this pathology added to the WHO classification of hematopoietic and lymphoid tumors in 1994. Like ALK, CD30 is a key factor triggering the pathogenic transformation cascade in ALCL. In the present study, CD30 expression was evaluated in all 100 patients and was found in 100% of cases (Figure 7).

Genetic rearrangements involving *ALK* are critical pathogenetic and diagnostic events in ALCL. Cytogenetic abnormalities were analyzed using fluorescence in situ hybridization (FISH). The characteristic t(2;5)(p23;q35) translocation, resulting in the *NPM–ALK* fusion transcript, was observed in ALK-positive ALCL cases. This chromosomal rearrangement was assessed in 10 patients (10%) and confirmed in all of them (100%).

Clinically, the most frequent manifestation at initial presentation was lymphadenopathy, observed in 94 patients (94%):

- Peripheral lymph nodes were involved in 64 patients (66%);
- Mediastinal lymph nodes—in 24 (25.6%);
- Abdominal lymph nodes—in 30 (30%).
- Internal organ involvement was less common:
- Intestinal involvement—in 30 patients (30%);
- Pulmonary and osseous involvement in 21 patients (21%).

Additionally:

- Tumor-associated pleural effusion was noted in four patients (4%);
- Malignant ascites in six patients (6%).

Specific tumor infiltration of soft tissues was observed in 32 patients (32%). The most common sites included:

- Soft tissues of the head—five patients (5%);
- Chest wall—12 patients (12%);
- Extremities—10 patients (10%).

Less common sites included:

- Neck—two patients (2%)
- Back—three patients (3%)

The clinical manifestations of the disease are summarized in Table 4.

**Figure 8:**
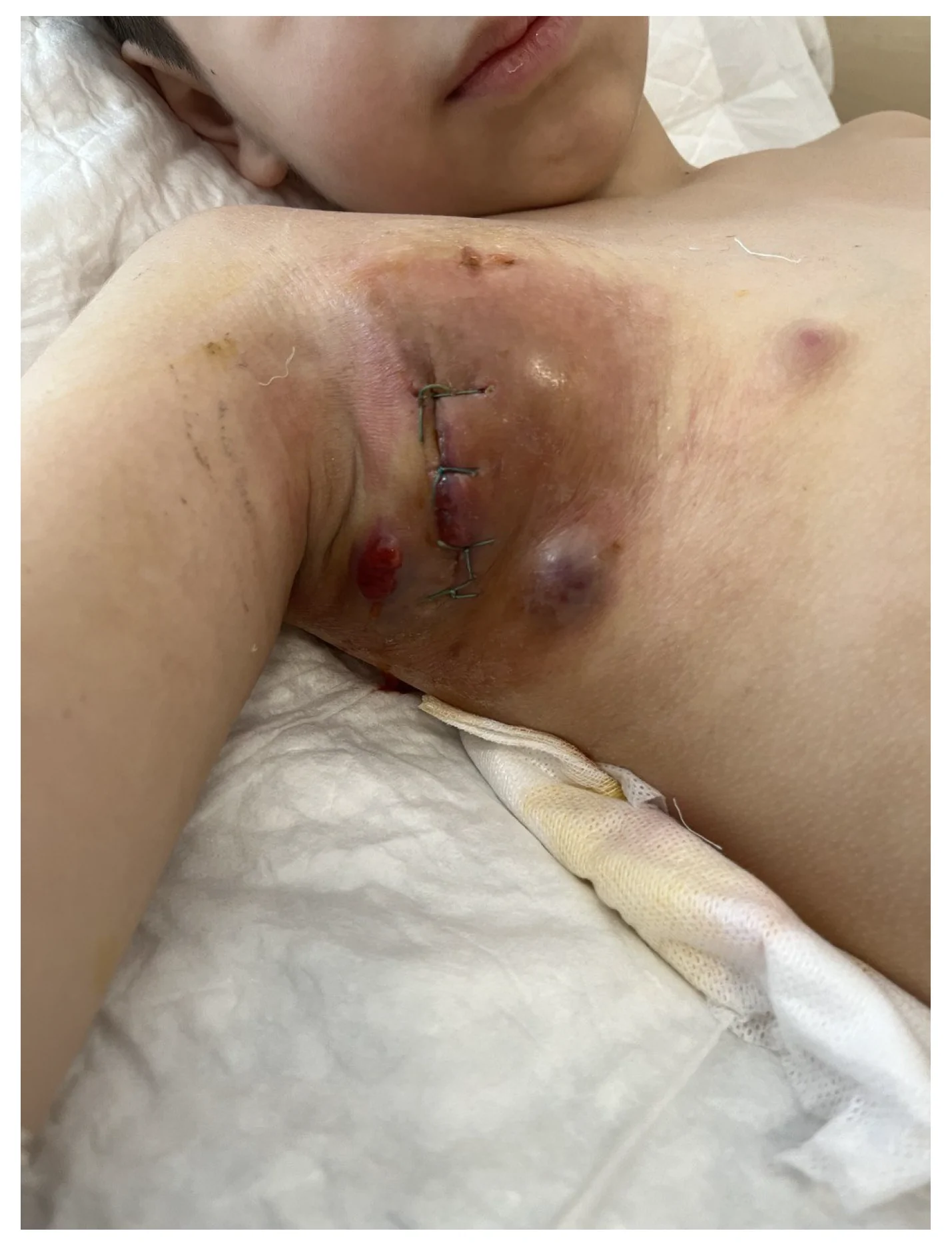
Skin lesion of the axillary region on the right during ALCL (own clinical case).

**Figure 9:**
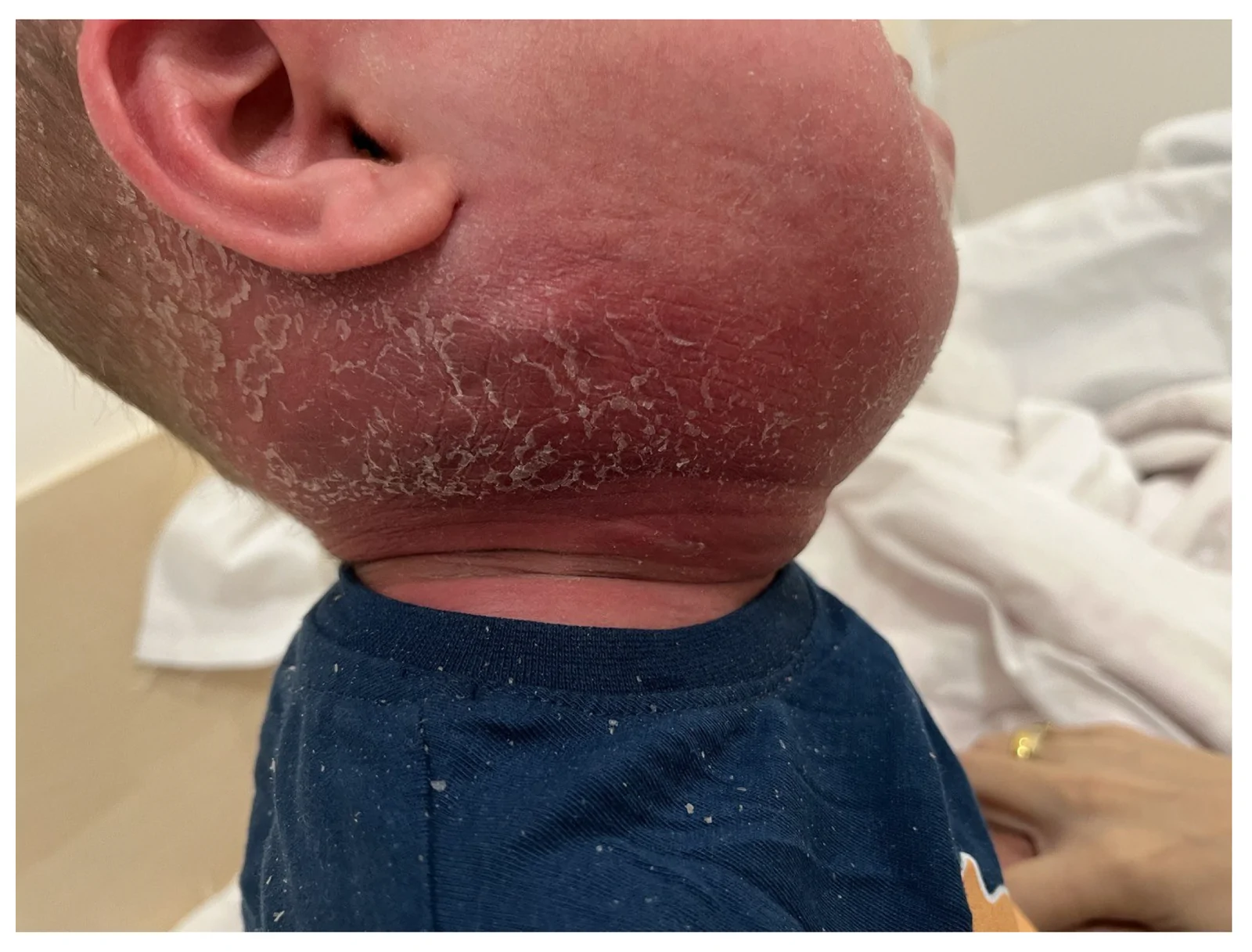
Skin lesions of the behind-the-ear, buccal, and submandibular regions and neck in ALCL (own clinical case).

**Figure 10:**
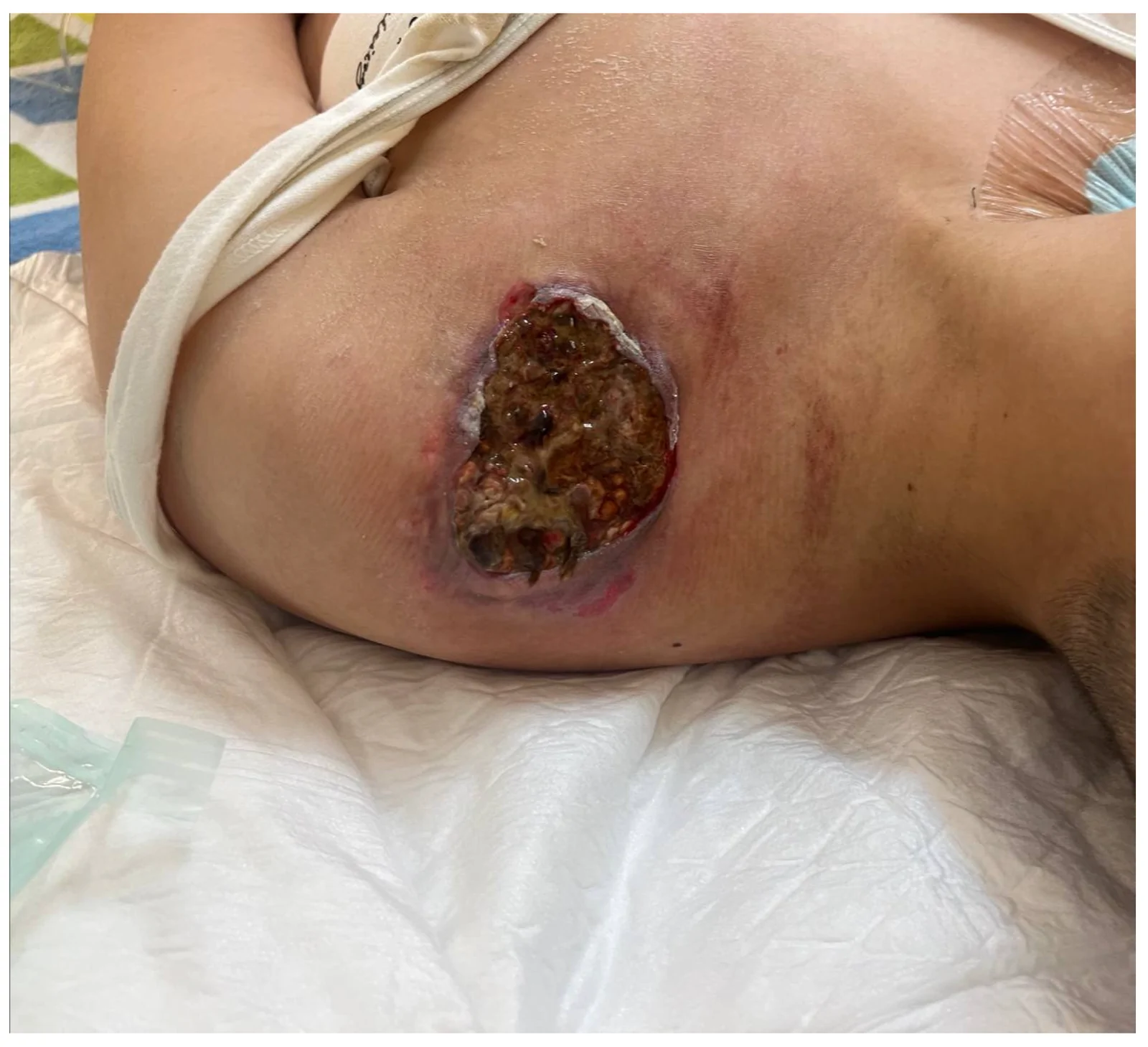
Damage to the skin with ulceration of the upper arm on the left with ALCL (own clinical case).

Skin involvement was identified in 12 cases (12%). Morphological skin lesions were mostly infiltrative, appearing as dense plaques that progressed to tumorous infiltrates with ulceration in 11 patients. Skin involvement was represented as nodular formations on the scalp in one patient (Figure 8, Figure 9 and Figure 10).

The rarest sites of ALCL involvement were bone marrow, in three patients (3%), and central nervous system (CNS) in one patient (1%). The patient with CNS involvement also had tumor cells in the bone marrow. According to the literature, involvement of these sites is associated with poor prognosis. In one patient (1%), the initial manifestation of disease was obstructive jaundice caused by compression of the common bile duct from a tumor conglomerate, which led to hyperbilirubinemia (the direct fraction) of up to 200 µmol/L. Based on the data obtained, the cohort of ALCL patients demonstrated clinical heterogeneity with involvement of both nodal and extranodal sites.

The aggressive nature of ALCL, including frequent involvement of organs relevant for risk stratification (skin, lungs, bones), resulted in the majority of patients being classified into the high-risk group: 76.9% in group A and 66.6% in group B.

Survival was evaluated across two groups of patients, based on treatment protocols.

**Figure 11:**
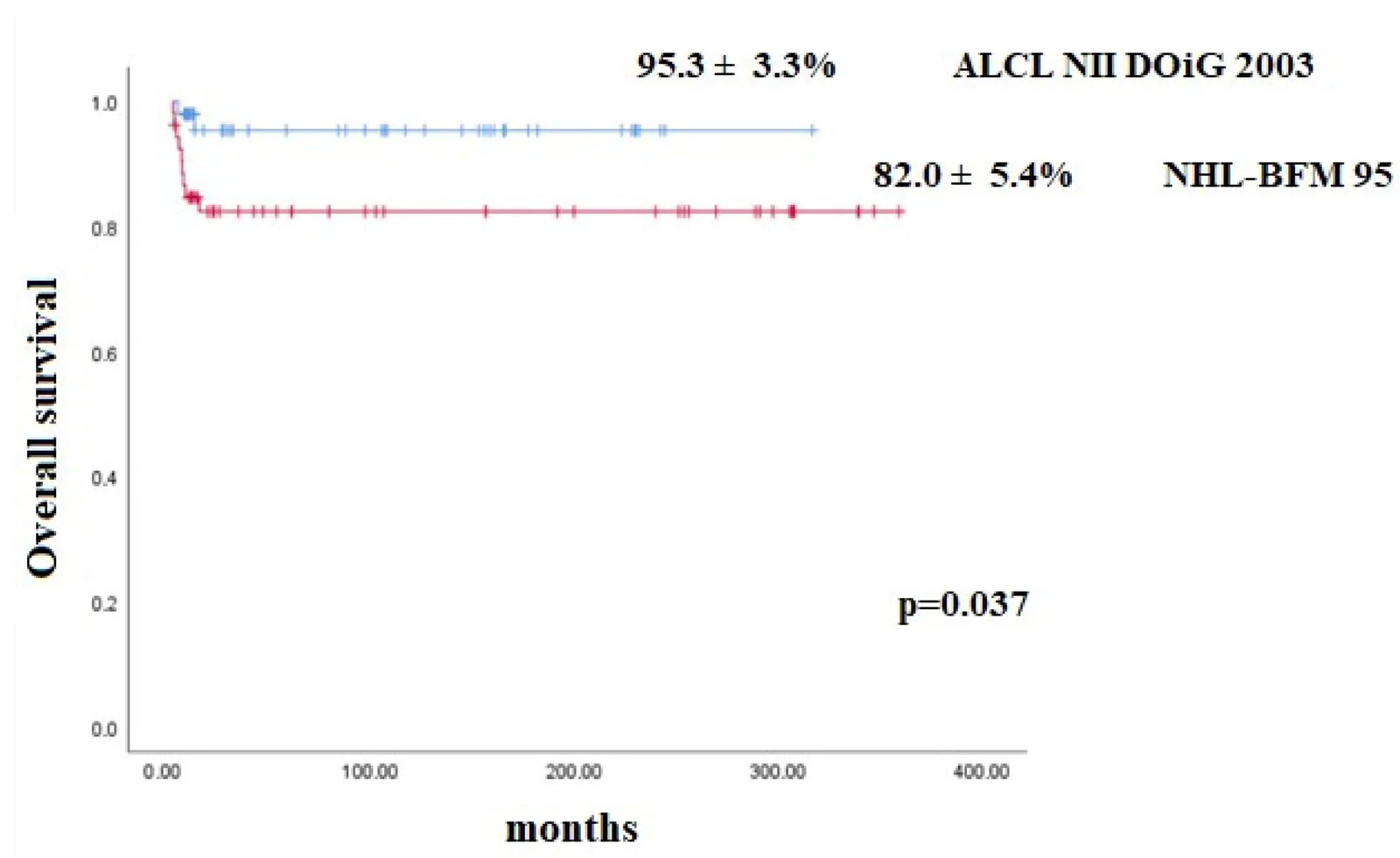
Overall survival of patients with anaplastic large cell lymphoma treated according to the ALCL NII DOG 2003 and NHL-BFM 95 protocols.

Statistically significant differences were identified, indicating superior efficacy of the ALCL NII DOiG 2003 protocol compared to the standard NHL-BFM 95 protocol (Figure 11).

The 10-year OS was as follows:

- ALCL NII DOiG 2003: 95.3 ± 3.3%;
- NHL-BFM 95: 82.0 ± 5.4%;
- *p* = 0.037.

The number of patients at risk and Kaplan–Meier estimates for OS at key time points are presented in Table 5. The decrease in the number of patients at risk over time might be primarily explained by censoring due to shorter follow-up in patients treated with the new protocol, as well as administrative censoring at the time of analysis.

The 5-year OS was similar to the 10-year OS, reflecting the early occurrence of events and subsequent plateau of the survival curves.

**Figure 12:**
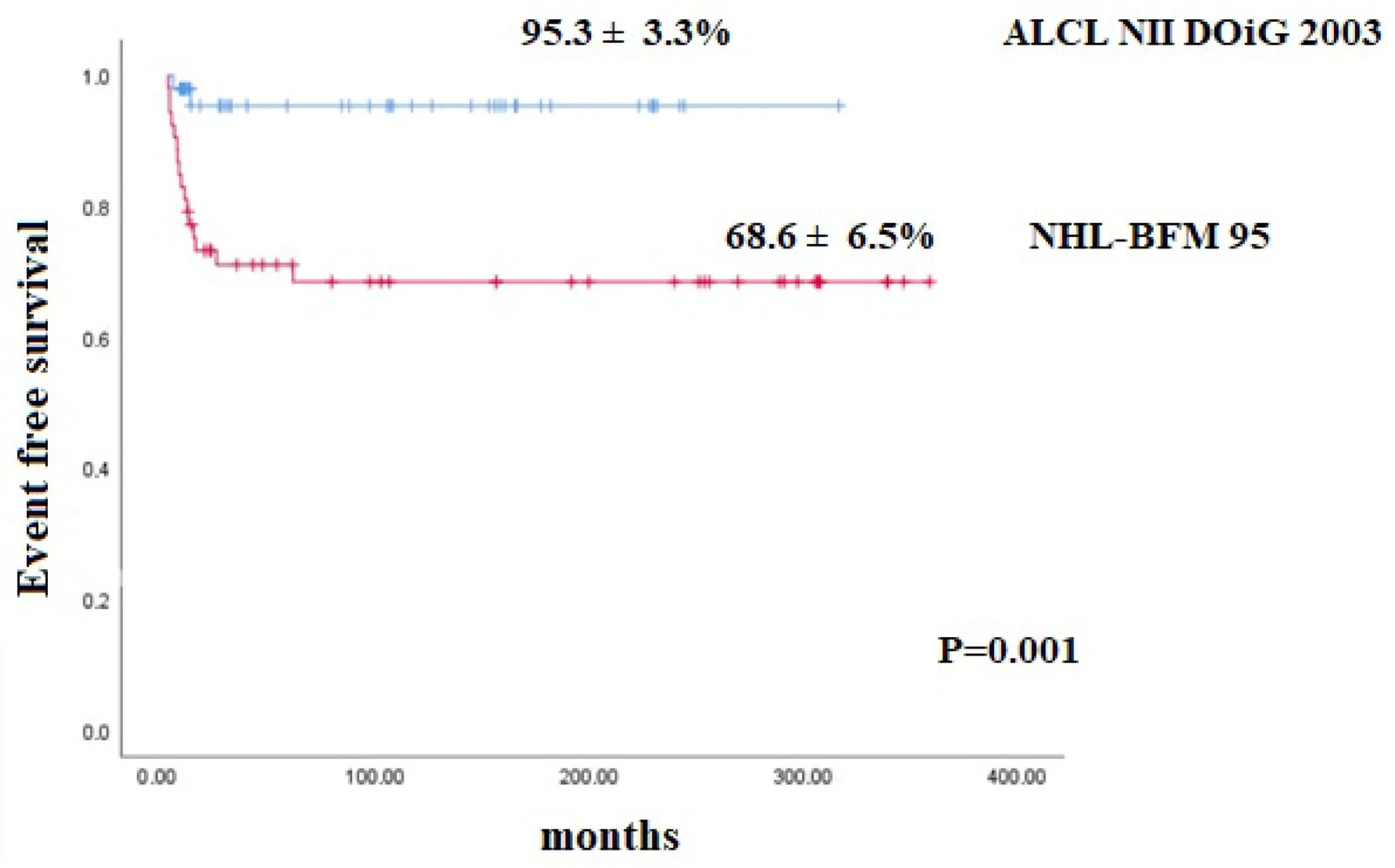
Event-free survival of patients with anaplastic large cell lymphoma treated according to the ALCL NII DOiG 2003 and NHL-BFM 95 protocols.

The 10-year EFS was significantly higher in patients treated with the ALCL NII DOiG 2003 protocol than in those treated with the NHL-BFM 95 protocol (Figure 12):

- 95.3 ± 3.3% vs. 68.6 ± 6.5%, respectively (*p* = 0.001).

The number of patients at risk and Kaplan–Meier estimates for EFS at key time points is presented in Table 6.

The data presented in the graph (Figure 12) demonstrate the higher efficacy of the ALCL NII DOiG 2003 protocol.

Only two events occurred with the treatment approach based on the immunological characteristics of the tumor.

In contrast, 16 events were recorded in patients treated with the NHL-BFM 95 protocol including one case of induction-related mortality (Table 7).

Among patients treated with the ALCL NII DOiG 2003 protocol, two out of 48 (4.1%) experienced adverse events (Table 8):

- one case of refractory disease;
- one case of partial response with subsequent progression.

**Figure 13:**
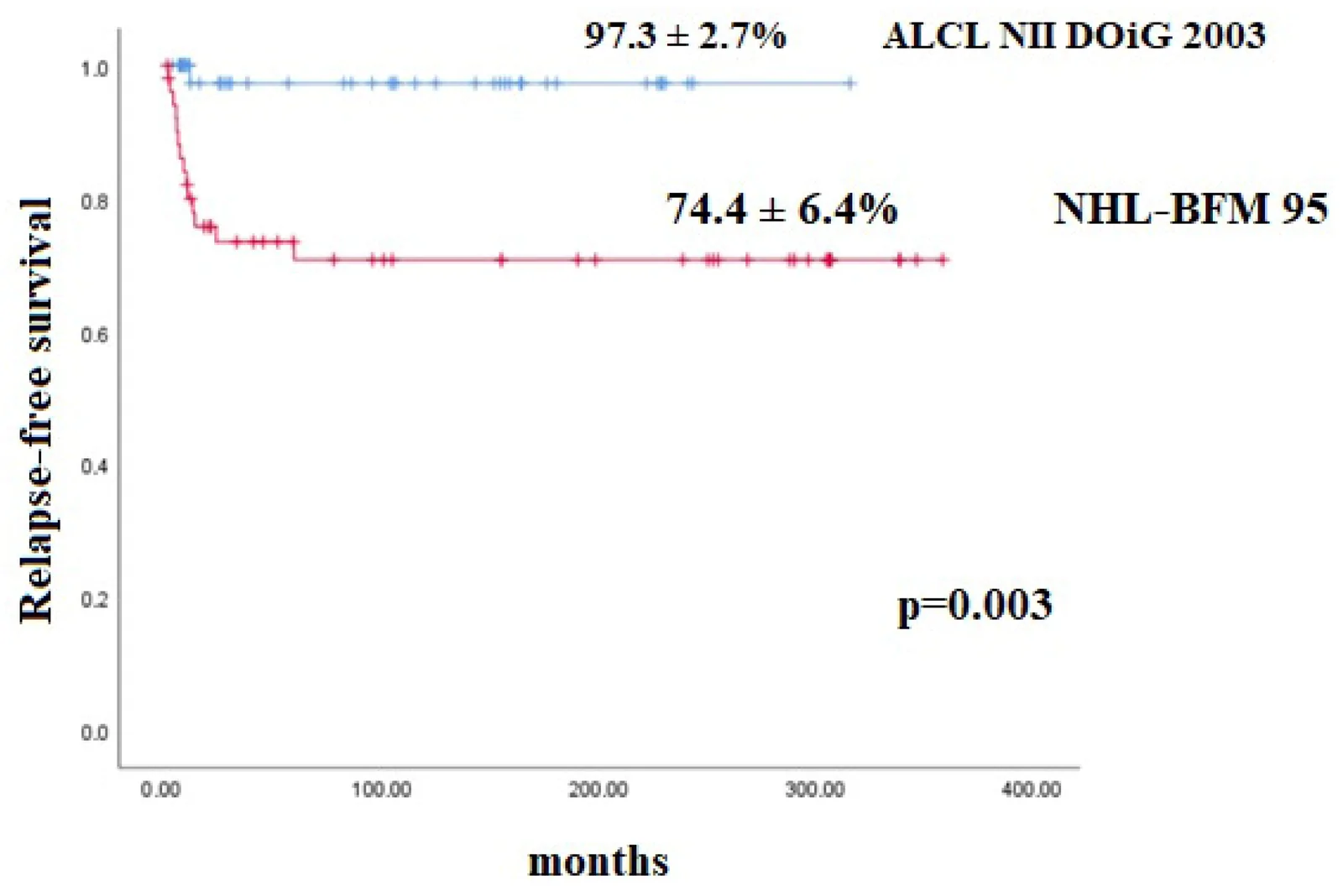
Relapse-free survival of patients with anaplastic large cell lymphoma treated according to the ALCL NII DOiG 2003 and NHL-BFM 95 protocols.

The 10-year RFS (Figure 13) was as follows:

- 97.3 ± 2.7% for patients treated with ALCL NII DOiG 2003;
- 74.4 ± 6.4% for those treated with NHL-BFM 95 (*p* = 0.003).

The number of patients at risk and Kaplan–Meier estimates for RFS at key time points are presented in Table 9.

Relapses were observed in eight patients (Table 10). In seven out of eight cases, relapses occurred within the first 12 months of the start of therapy and were classified as early relapses. Only one patient experienced a late relapse. Six deaths occurred among relapsed patients, four due to infectious complications and two due to disease progression.

A comparative analysis of survival outcomes was performed according to risk stratification (Table 11).

Findings demonstrated that:

- Patients treated with the ALCL NII DOiG 2003 protocol achieved survival rates exceeding 90% in all risk groups.
- Patients treated with the standard NHL-BFM 95 protocol, particularly in the standard and intermediate risk groups, also showed good survival outcomes.
- The majority of adverse events occurred among patients in the high-risk group.

Given the unfavorable prognostic value of T-cell marker expressions, survival analysis was performed based on lymphoma immunophenotype (Table 12).

## 4. Discussion

Anaplastic large cell lymphoma is a highly aggressive subtype of non-Hodgkin lymphoma, accounting for 10–15% of all pediatric NHL cases. ALCL is characterized by clinical, morphological, and immunological heterogeneity, including variable expression of T-cell markers [1,2,3].

Despite controversial data on the prognostic significance of T-cell marker expressions, most clinical studies showed that patients with CD3-positive ALCL had poorer outcomes than those with CD3-negative ALCL. In particular, Muzzafar et al. and Abramov et al. demonstrated the adverse prognostic impact of CD8 and CD3 expression by tumor cells [14,15]. These findings showed the importance of the stratification of ALCL patients according to T-cell markers. In the present study, we performed a comparative evaluation of the efficacy of the NHL-BFM 95 protocol, which did not include T-cell marker detection, and a new immunophenotype-oriented treatment protocol (ALCL NII DOiG 2003) that involved lymphoma immunologic characteristics.

According to the literature, a variety of treatment approaches are used for ALCL, including intensive block-based regimens and protocols similar to those used in treating ALL. Despite differences in treatment duration and chemotherapy agents, no clear advantage has been shown for any particular therapy. Reported OS and EFS rates reach 75–80%, regardless of the chosen therapeutic strategy. Thus, the 5-year EFS in patients with ALCL treated according to the NHL-BFM 90 protocol reached 76% [20]. The use of the ALL-oriented POG APO 9315 protocol also did not lead to an increase in survival rates: the 4-year EFS and OS were 71.8% and 88.1% [22].

It is important to emphasize that the literature review showed the lack of studies that evaluate expression of T-cell markers when selecting treatment for ALCL.

The ALCL NII DOiG 2003 protocol was designed taking into account the emerging insights into poor prognostic factors in ALCL. This protocol integrates not only the disease stage and risk group but also the immunophenotypic characteristics of the tumor.

The results demonstrated the distinct benefit of the immunophenotype-oriented approach:

- 10-year OS was 95.3 ± 3.3% in Group 1 (treated with ALCL NII DOiG 2003) vs. 82.0 ± 5.4% in Group 2 (treated with NHL-BFM 95), *p* = 0.037;
- 10-year EFS: 95.3 ± 3.3% vs. 68.6 ± 6.5% in Group 1 and Group 2, respectively, *p* = 0.001;
- 10-year RFS: 97.3 ± 2.7% vs. 74.4 ± 6.4% in Group 1 and Group 2, respectively, *p* = 0.003.

Given the high survival rates achieved with chemotherapy alone, the intensification of first-line therapy through the addition of immunoagents is currently not warranted, as confirmed by the results of the ANHL12P1 trial [25,26]. Patients with ALCL stages II–IV received chemotherapy based on the ALCL99 protocol, with the inclusion of Brentuximab vedotin or Crizotinib in each treatment block. In the group treated with Brentuximab vedotin, a high two-year OS was achieved—97%; however, the two-year EFS was 79%, with relapses occurring in 14 of 67 patients. The results from the other treatment arm, which included Crizotinib, were comparable: the two-year EFS was 76.8%, and the two-year OS was 95.2%. Relapses occurred in 15 of 66 patients.

An important and pressing task for researchers remains the determination of optimal therapeutic strategies for relapsed or refractory ALCL. A gold standard for therapy has not yet been established; various regimens are used to achieve remission, ranging from high-dose chemotherapy to less intensive approaches, including immunotherapy. Allo-HSCT has become a necessary option for consolidation of remission, primarily in cases of refractory ALCL and early relapses, which was supported by the data from multiple research groups, including the BFM and EICNHL consortia [38,39].

ALCL tumor phenotype has long been an area of interest for researchers; for instance, back in 1993, Vecchi et al. particularly indicated the expression of T-cell antigens on tumor cells when describing the results of ALCL treatment in children, though the prognostic value of this marker was not considered [40]. The immune-oriented protocol was studied based on the available literature data, as well as the authors’ own clinical experience that supported the results of the negative prognostic value of T-cell marker expression in ALCL [41]. Modification of ALCL therapy according to the immunological features of the tumor could show benefits for the outcomes in the treatment of children with ALCL.

This study has several limitations. Firstly, the retrospective–prospective design, along with the lack of randomization and open-label design, may introduce potential selection and treatment biases. Secondly, a relatively small sample size and imbalance between the treatment groups—particularly in the high-risk cohort, where more patients were treated according to the NHL-BFM 95 protocol compared to the new protocol (40 vs. 32 patients)—may affect the robustness of the comparative analysis. Thirdly, although the median follow-up was 10 years, not all patients reached that long-term follow-up, and survival estimates were based on the Kaplan–Meier curves with censored observations. Finally, several potentially important prognostic factors, including ALK expression, minimal disseminated disease status, t(2;5) translocation, and the lymphohistiocytic histological subtype were not evaluated, which may limit the interpretation of survival outcomes. Further studies could consider these limitations in their research.

## 5. Conclusions

Thus, the treatment outcomes for children with ALCL under the differentiated, immunologically guided protocol ALCL NII DOiG 2003 were significantly superior to those achieved with the standard treatment protocol. The use of the ALCL NII DOiG 2003 protocol allowed us to achieve a 10-year OS = 95.3 ± 3.3%, EFS = 95.3 ± 3.3%, and RFS = 97.3 ± 2.7%. Treatment of children with ALCL by the standard NHL-BFM 95 protocol, which does not consider the immunological features of the tumor, with a similar follow up period resulted in significantly lower outcomes such as OS = 82.0 ± 5.4%, EFS = 68.6 ± 6.5%, and RFS = 74.4 ± 6.4%.

The differentiated, immune-oriented, and risk-adapted therapeutic approach achieved high survival rates and a reduction in refractory disease and relapses, even in cases with commonly unfavorable prognostic factors, such as expression of T-cell markers and advanced ALCL with involvement of high-risk organs (skin, bones, lungs). The results of the research were introduced into practice in Eurasian and Russian clinical settings and are successfully used for the treatment of children with ALCL.

In most cases, ALCL is ALK-positive and CD30-positive; in our study, CD30 was detected in all patients and ALK in 98%. The use of targeted monoclonal-antibody-based agents against ALK and CD30 is undoubtedly a promising therapeutic strategy for ALCL. However, no clear indications have currently been adopted for such a therapy. These targeted agents can be recommended as second-line therapy when standard regimens are ineffective in patients with relapsed diseases. The rationale for using CD30 CAR-T cells is even less certain. Despite the revolutionary advances of CAR-T therapy in B-cell lymphomas, the limited clinical studies of CD30 CAR-T cells have yielded contradictory results, preventing an assessment of the promise of this approach as adoptive immunotherapy for ALCL.

ICI therapy in patients with ALCL has shown rather modest efficacy; moreover, in some cases hyperprogression of the disease was observed, which was interpreted as stimulation of lymphoma cells [29]. Combining ICIs with allo-HSCT increased clinical efficacy, mainly due to enhancement of GVHD and the graft-versus-lymphoma effect. However, this combination is poorly tolerated, and in several cases ICIs were discontinued because of severe adverse events [29]. High-dose chemotherapy followed by auto-HSCT proved to be of limited efficacy, in contrast to allo-HSCT, whose clinical effect has been attributed to the aforementioned graft-versus-lymphoma phenomenon.

The present clinical study showed that the immuno-oriented treatment achieved a 100% clinical response, and the probability of relapse over 10 years was 1%. The ALCL NII DOiG 2003 protocol demonstrated acceptable tolerability and did not require HSCT.

Given the high efficacy of the ALCL NII DOiG 2003 protocol, additional immunotherapy is not justified. However, targeted agents and CAR-T cells may be considered in the treatment of refractory/relapsed ALCL.

A stratified chemotherapy that considers the immunological characteristics of the tumor has led to significant advances in the outcomes of ALCL patients, regardless of the unfavorable prognostic factors.

## Figures and Tables

**Table 1:** Differential diagnostic immunohistochemical characteristics of ALK+ ALCL.

Characteristic Markers	Non-Characteristic Markers
CD30 (cytoplasmic), EMA, CD2, CD4, CD5, TIA1, granzyme B, perforin, CD45, CD45RO, CD61, CD25, BNH9	CD15, CD20, CD79a, cytokeratin, bcl2, PAX5/BSAP, PGM1, EBV (EBER and LMP1)

**Table 2:** Doses of chemotherapy drugs for intrathecal administration, by patient age.

Age of Patients	Methotrexate	Cytarabine	Prednisolone
under 1 year old	6 mg	16 mg	4 mg
from 1 to 2 years old	8 mg	20 mg	6 mg
from 2 to 3 years old	10 mg	26 mg	8 mg
3 years and older	12 mg	30 mg	10 mg

**Table 3:** Comparative characteristics of patients with ALCL by the treatment protocol.

Parameter	Protocol	
	NHL-BFM 95	ALCL NII DOG 2003
Number of patients	52	48
Age (median), years	11 (2–17)	11 (4–18)
Boys:girls	1.6:1	1.36:1
Stage I	0	3 (6.2%)
Stage II	8 (15.3%)	10 (20.8%)
Stage III	31 (59.6%)	26 (54%)
Stage IV	13 (25%)	9 (18.7%)
Standard risk group	0 (0%)	1 (2%)
Intermediate risk group	12 (23%)	15 (31.25%)
High risk group	40 (76.9%)	32 (66.6%)

**Table 4:** Clinical manifestations of ALCL.

Localization	Number of Patient (Total *n* = 100)	
	Abs. *n*	%
Skin	12	12
Lymph nodes	94	94
Salivary gland	2	2
Spleen	4	4
Intestine	30	30
Liver	5	5
Pancreas	7	7
Kidneys	8	8
Central nervous system	1	1
Lungs	21	21
Bone marrow	3	3
Soft tissues	32	32
Bones	21	21

**Table 5:** Number of patients at risk and Kaplan–Meier estimates for overall survival at 0, 60 and 120 months.

Time (Months)	NHL-BFM 95, Number of Patients at Risk	NHL-BFM 95, OS, 95% CI	ALCL NII DOiG 2003, Number of Patients at Risk	ALCL NII DOiG 2003, OS, 95% CI
0	52	0.981 (0.944–1.000)	48	1.000
60	48	0.82 (0.714–0.926)	40	0.953 (0.92–0.986)
120	45	0.82 (0.714–0.926)	40	0.953 (0.92–0.986)

OS = overall survival, CI = confidence interval.

**Table 6:** Number of patients at risk and Kaplan–Meier estimates for event-free survival at 0, 60 and 120 months.

Time (Months)	NHL-BFM 95, Number of Patients at Risk	NHL-BFM 95, EFS, 95% CI	ALCL NII DOiG 2003, Number of Patients at Risk	ALCL NII DOiG 2003, EFS, 95% CI
0	52	0.962 (0.911–1.000)	48	1.000
60	45	0.686 (0.621–0.751)	45	0.953 (0.92–0.986)
120	41	0.686 (0.621–0.751)	45	0.953 (0.92–0.986)

EFS = event-free survival, CI = confidence interval.

**Table 7:** Events in patients treated with the ALCL NII DOiG 2003 and NHL-BFM 95 protocols.

Event	NHL-BFM 95	ALCL NII DOiG 2003
Progression	2	0
Refractory disease	1	1
Relapse	12	1
Induction-related mortality	1	0

**Table 8:** Characteristics of patients with relapsed/refractory ALCL treated with the ALCL NII DOiG 2003 protocol.

Patients	Histological Variant and Immunophenotype of Tumor	Response to 1st-Line Therapy	2nd-Line and Subsequent Lines of Therapy	Outcome
1	Cassic variant with expression of T-cell markers	Refractory disease, disease progression	VBL, HD MTX, BV; CZ	Death
2	Small-cell variant with expression of T-cell markers	Partial response, disease progression	ViGEPD + BV (4) + alloHSCT VBL + CZ	Death

Note: VBL—Vinblastine, HD MTX—High-dose methotrexate, BV—Brentuximab vedotin, CZ—Crizotinib, ViGEPD + BV—Vinblastine, gemcitabine, dacarbazine, Brentuximab vedotin, HSCT—Hematopoietic stem cell transplantation.

**Table 9:** Number of patients at risk and Kaplan–Meier estimates for relapse-free survival at 0, 60 and 120 months.

Time (Months)	NHL-BFM 95, Number of Patients at Risk	NHL-BFM 95, EFS, 95% CI	ALCL NII DOiG 2003, Number of Patients at Risk	ALCL NII DOiG 2003, EFS, 95% CI
0	52	1.000	48	1.000
60	45	0.744 (0.68–0.80)	46	0.973 (0.946–0.986)
120	41	0.744 (0.68–0.80)	46	0.973 (0.946–0.986)

EFS = event-free survival, CI = confidence interval.

**Table 10:** Characteristics of patients with ALCL relapse treated with the NHL-BFM 95 protocol.

Patients	Early/Late Relapse	2nd and Subsequent Lines of Therapy	Outcome
1	Early	Radiotherapy, HR1	Death
2	Early	HAM	Death
3	Late	VBL, MTX, etoposide, 6-MP	Alive
4	Early	CycloVPCarb + FLU Ara-C + radiotherapy	Death
5	Early	HD MTX + VBL, BV + ICE, autoHSCT, BV + ViGEPD + CZ, alloHSCT	Alive
6	Early	ICE	Death
7	Early	VIGEPD + BV	Death
8	Early	CycloVPCarb	Death

Note: HR1—Vincristine, methotrexate (5000 mg/m^2^), dexamethasone, cytarabine, cyclophosphamide, L-asparaginase, HAM—Cytarabine, mitoxantrone, 6-MP—6-mercaptopurine, CycloVPCarb—Cyclophosphamide, etoposide, carboplatin, HD MTX—High-dose methotrexate, VBL—Vinblastine, BV—Brentuximab vedotin, ICE—Ifosfamide, carboplatin, etoposide, FLU Ara-C—Fludarabine, cytarabine, ViGEPD—Vinblastine, gemcitabine, dacarbazine, prednisolone, CZ—Crizotinib, HSCT—Hematopoietic stem cell transplantation.

**Table 11:** Comparative analysis of treatment effectiveness in ALCL patients by risk group.

Parameter	*p*-Value	Protocol	
		NHL-BFM 95	ALCL NII DOiG 2003
Standard risk group			
OS	N/A	N/A	100%
EFS	N/A	N/A	100%
RFS	N/A	N/A	100%
Intermediate risk group			
OS		100%	100%
EFS	0.34	90.9 ± 8.7%	100%
RFS	0.34	90.9 ± 8.7%	100%
High risk group			
OS	0.069	74.6 ± 7.7%	93.0 ± 4.8%
EFS	0.005	59.7 ± 8.3%	93.0 ± 4.8%
RFS	0.004	62.9 ± 8.4%	96.0 ± 3.9%

Note: N/A = not available.

**Table 12:** Comparative analysis of treatment effectiveness in ALCL patients by lymphoma immunophenotype.

Parameter	*p*-Value	Protocol	
		NHL-BFM 95	ALCL NII DOiG 2003
T^+^			
OS	0.053	86.7 ± 6.8%	91.2 ± 6%
EFS	0.001	68.8 ± 11.6%	91.2 ± 6%
RFS	0.001	73.3 ± 11.4%	94.7 ± 5.1%
T^−^			
OS	0.041	80 ±6.8%	100%
EFS	0.001	68.8 ±7.8%	100%
RFS	0.001	70.8 ±7.8%	100%

Note: T^+^ = expression of T-cell markers; T^−^ = no expression of T-cell markers.

## Data Availability

The datasets presented in this article are not readily available due to privacy regulations but are available from the corresponding author upon reasonable request. All data shared will be de-identified in accordance with ethical guidelines.

## Abbreviations

The following abbreviations are used in this manuscript:

ALCL	anaplastic large cell lymphoma
ALK	anaplastic lymphoma kinase
ALK−	ALK-negative
ALK+	ALK-positive
allo-HSCT	allogeneic hematopoietic stem cell transplantation
auto-HSCT	autologous hematopoietic stem cell transplantation
BFM	Berlin–Frankfurt–Münster group
CAR	chimeric antigen receptor
CCG	Children’s Cancer Group
CNS	central nervous system
COG	children’s oncology group
EFS	event-free survival
FDA	Food and Drug Administration
FISH	fluorescence in situ hybridization
GVHD	graft-versus-host disease
ICIs	immune checkpoint inhibitors
IU	international units
IV	intravenous
MTX	methotrexate
NHL	non-Hodgkin lymphoma
ORR	objective response rate
OS	overall survival
PD-1	programmed cell death 1
PD-L1	programmed death-ligand 1
POG	Pediatric Oncology Group
r/r	relapsed/refractory
RFS	relapse-free survival
TNFR	tumor necrosis factor receptor
WHO	World Health Organization
