# Correction: Volkova et al. Anaplastic Large Cell Lymphoma in Children: 20-Year Immune-Oriented Treatment Experience. *Cancers* 2026, *18*, 1583

**DOI:** 10.3390/cancers18203337

**Published:** 2026-10-16

**Authors:** Anastasiya S. Volkova, Timur T. Valiev, Mikhail V. Kiselevskiy, Irina Zh. Shubina, Kirill I. Kirgizov, Svetlana R. Varfolomeeva, Ivan S. Stilidi

**Affiliations:** 1 Research Institute of Pediatric Oncology and Hematology, FSBI “N.N. Blokhin National Medical Research Center of Oncology” of the Ministry of Health of Russia, Kashirskoye sh. 23, Moscow 115522, Russia; a.s.volkova@ronc.ru (A.S.V.); t.valiev@ronc.ru (T.T.V.); k.kirgizov@ronc.ru (K.I.K.); s.varfolomeeva@ronc.ru (S.R.V.); 2 Research Institute of Experimental Oncology and Carcinogenesis, FSBI “N.N. Blokhin National Medical Research Center of Oncology” of the Ministry of Health of Russia, Kashirskoye sh. 24, Moscow 115522, Russia; kisele@inbox.ru; 3 Management Department, FSBI “N.N. Blokhin National Medical Research Center of Oncology” of the Ministry of Health of Russia, Kashirskoye sh. 24, Moscow 115522, Russia; director@ronc.ru

## Text Correction

The following corrections have been made to our original published paper [1]:

Section 2, Materials and Methods, Paragraph 12, incorrect:

- Risk Group I: Treatment consisted of 3 blocks (alternating 1a #2, 1b#1) administered every two weeks.
- Risk Group II: Patients received 6 blocks of polychemotherapy (1a #3, 1b #3) administered at two-week intervals.

Corrected:

- Risk Group I (standard risk): Treatment consisted of 3 blocks (alternating 1a #2, 1b#1) administered every two weeks.
- Risk Group II (intermediate risk): Patients received 6 blocks of polychemotherapy (1a #3, 1b #3) administered at two-week intervals.

Paragraph 15, incorrect: “Treatment for Risk Group III:”

Corrected: “Treatment for Risk Group III (high risk):”

Section 3, Results, the captions of Figures 8–10, incorrect:

- **Figure 8.** Skin lesion of the axillary region on the right during ALCL (own clinical observation).
- **Figure 9.** Skin lesions of the behind-the-ear, buccal, and submandibular regions and neck in ALCL (own clinical observation).
- **Figure 10.** Damage to the skin with ulceration of the upper arm on the left with ALCL (own clinical observation).

Corrected:

- **Figure 8.** Skin lesion of the axillary region on the right during ALCL (own clinical case).
- **Figure 9.** Skin lesions of the behind-the-ear, buccal, and submandibular regions and neck in ALCL (own clinical case).
- **Figure 10.** Damage to the skin with ulceration of the upper arm on the left with ALCL (own clinical case).

The authors state that the scientific conclusions are unaffected. This correction was approved by the Academic Editor. The original publication has also been updated.
